# Supplementary material for: Drug repurposing for aging research using model organisms
Source: Aging Cell. 2017 Jun 16;16(5):1006–15. doi: 10.1111/acel.12626 (PMC5595691; doi:10.1111/acel.12626)
Supplement: Supplementary file 7 — Data S1 Zip‐Archive of all report cards. [file ACEL-16-1006-s007.zip › RC_B21.pdf]

B21

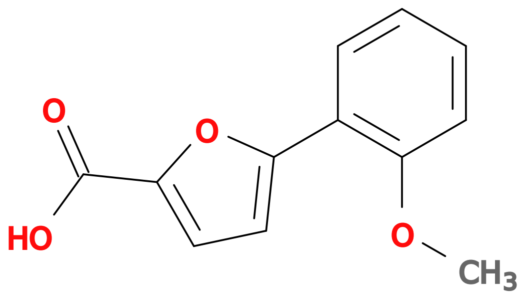

Database identifiers

ChEMBLCompound	CHEMBL372832
DrugBank	DB07407
eMolecules	812950

Ranking

	Rank	Score
Drosophila	532/697	0.223
C. elegans	299/591	0.192

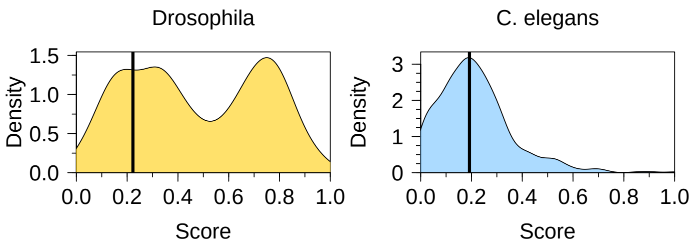

	Ageing implication	Domain conservation	Binding site conservation	Binding affinity	Bioavailability	Lipinski	Promiscuity	Purchasability	Drug approval	**Total**
Drosophila	0.203	0.911	1.0	0.291	(0.9)	0.0	-0.0	0.1	0.075	0.223
C. elegans	0.203	0.903	1.0	0.291	0.331	0.0	-0.0	0.1	0.075	0.192

Names

No synonyms found

Roles

ChEBI entry None has no roles

Status

Approved drug (according to ChEMBL)	No
Classification (according to DrugBank)	experimental
Number of Rule of 5 violations	0
Binding affinity to original target in log units (RF-Score prediction)	4.11
Burns *C. elegans* bioavailability prediction	0.33

Compound Target Characteristics

Peptidyl-prolyl cis-trans isomerase NIMA-interacting 1

Best gene implication in ageing for this target family came from gene Q13526 via mapping the annotation from Ensembl ENSG00000127445 via mapping the annotation from EntrezGene 5300 via mapping the annotation from GenAgeHuman 0062 annotated in GenAge release 17.

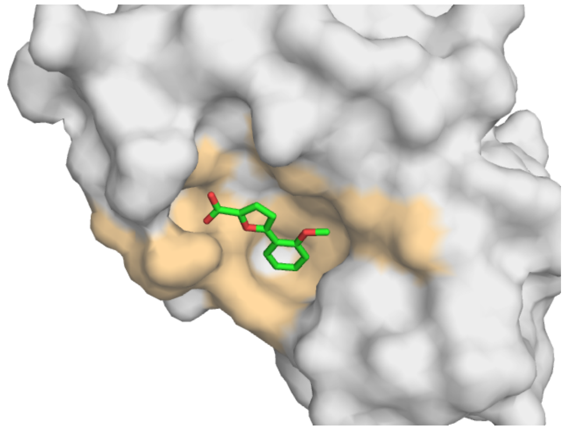

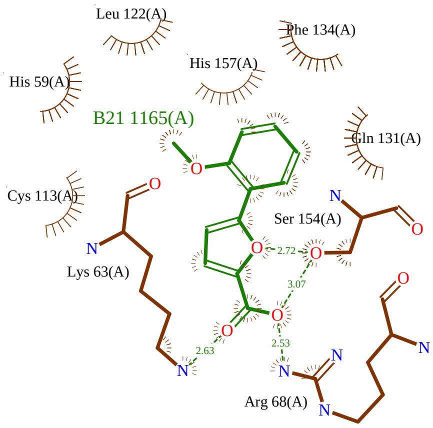

protein	amino acids contacts (binding site)
PDB:2xp3:chainA:Q13526	H K R C L Q F S H
sp:Q13526:PIN1_HUMAN	H K R C L Q F S H
tr:B0BNL2:B0BNL2_RAT	H K R C L Q F S H
sp:Q9QUR7:PIN1_MOUSE	H K R C L Q F S H
sp:P54353:DOD_DROME	H K R C L Q F S H
tr:Q9N492:Q9N492_CAEEL	H K R C L Q F S H
sp:P22696:ESS1_YEAST	H K R C L Q F S H

	whole protein		domain-based		contact-based	
protein	ident	simil	ident	simil	ident	simil
PDB:2xp3:chainA:Q13526	0.99	1.0	1.0	1.0	1.0	1.0
sp:Q13526:PIN1_HUMAN	1.0	1.0	1.0	1.0	1.0	1.0
tr:B0BNL2:B0BNL2_RAT	0.96	0.98	0.99	1.0	1.0	1.0
sp:Q9QUR7:PIN1_MOUSE	0.95	0.98	0.97	1.0	1.0	1.0
sp:P54353:DOD_DROME	0.55	0.82	0.58	0.83	1.0	1.0
tr:Q9N492:Q9N492_CAEEL	0.54	0.81	0.55	0.82	1.0	1.0
sp:P22696:ESS1_YEAST	0.44	0.78	0.46	0.8	1.0	1.0
